# Mesoporous TiO_2_–SiO_2_ adsorbent for ultra-deep desulfurization of organic-S at room temperature and atmospheric pressure†

**DOI:** 10.1039/c8ra00112j

**Published:** 2018-02-16

**Authors:** Bin Qin, Yuesong Shen, Boyang Xu, Shemin Zhu, Peiwen Li, Youlin Liu

**Affiliations:** College of Materials Science and Engineering, Nanjing Tech University No. 5 Xinmofan Road Nanjing 210009 China sys-njut@163.com; Jiangsu Collaborative Innovation Center for Advanced Inorganic Function Composites, Nanjing Tech University No. 5 Xinmofan Road Nanjing 210009 China; Jiangsu National Synergetic Innovation Center for Advanced Materials, Nanjing Tech University No. 5 Xinmofan Road Nanjing 210009 China; Department of Aerospace and Mechanical Engineering, The University of Arizona Tucson AZ 85721-0119 USA peiwen@email.arizona.edu

## Abstract

Ultra-deep desulfurization is a major requirement for upgrading the quality of fuel and power sources for fuel-cells. A series of mesoporous TiO_2_–SiO_2_ adsorbents were prepared and investigated for ultra-deep adsorption of benzothiophene (BT) and dibenzothiophene (DBT) from model fuel at ambient conditions. The adsorbents were characterized *via* SEM, XRD, N_2_-BET, FT-IR and NH_3_-TPD techniques. The results revealed that the adsorbent containing 40 wt% silica achieved the desulfurization efficiency higher than 99% when the initial sulfur concentration in the model fuel was 550 ppm. The high desulfurization performance of the adsorbent was attributed to its large specific surface and surface acidity. It also achieved a high sulfur adsorption capacity of 7.1 mg g^−1^ in a fixed-bed test, while its static saturated sulfur capacity was 13.7 mg g^−1^. The order of selectivity towards the adsorption of different organic sulfurs was DBT > BT&DBT > BT. The kinetics of the adsorption of organic sulfur was studied and the results indicated that the pseudo-second order model appropriately fitted the kinetics data. Furthermore, the used adsorbent can be easily regenerated and the desulphurization efficiency of the recovered adsorbent after five regeneration cycles was still maintained at 94.5%.

## Abstract 2

Ultra-deep desulfurization is a major requirement for upgrading the quality of fuel and power sources for fuel-cells.

## Graphical abstract

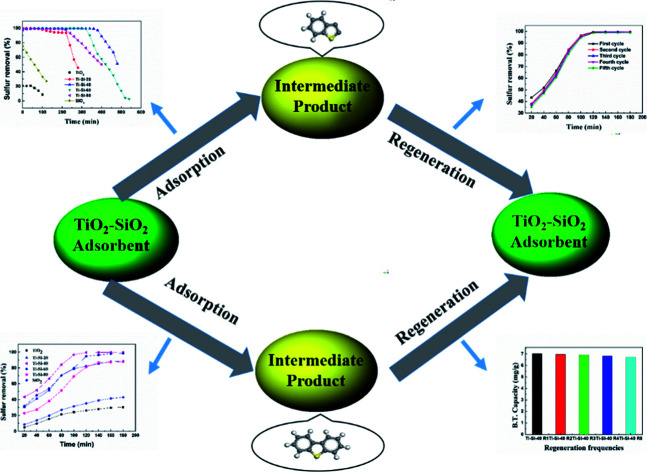

## 1. Introduction

Sulfur compounds in fuels are the main cause of air pollution, particularly resulting in acid rain. In addition, sulfur in fuels used for fuel-cell applications can poison the anodes and decline the efficiency of the fuel-cells. Thus, fuel-cell applications require fuels with ultra-low concentrations of sulfur compounds (<1 ppm for PEMFCs and <10 ppm for SOFCs).^1–3^ Therefore, it is important to develop ultra-deep desulfurization technologies to satisfy the rapid development of fuel-cells.

Deep desulfurization of traditional fuels has been explored in the past decades. Hydrodesulfurization (HDS) is a conventional process for removing sulfur from fossil fuels.^4^ HDS exhibits high efficiency in eliminating mercaptans, sulfides, disulfides and some derivatives of thiophene (T). However, several sulfur compounds such as benzothiophene (BT), dibenzothiophene (DBT) and their derivatives are hardly removed by HDS. Moreover, the operating temperature and pressure of HDS is very high, which makes its operation dangerous. In addition, the conversion rates of T, BT and DBT during HDS follows the order T > BT > DBT, while the conversion rate of 4,6-dimethyldibenzothiophene (4,6-DMDBT) is the lowest.^5^ In order to satisfy regulation requirements for protecting the environment, deep desulfurization technologies are necessary to further remove sulfur from fuels obtained after HDS.

Oxidative desulfurization (ODS) and extractive desulfurization (EDS) are new technologies for deep desulfurization that have become popular because of their mild operating conditions.^6–10^ Moreover, the sulfur compounds that remain after HDS can be effectively converted by utilizing other technologies. Thiophene and its derivatives were selectively oxidized into sulfones and/or sulfoxides by ODS. The sulfones and sulfoxides could easily be removed by extraction and adsorption processes because of their high polarity. Rivoira *et al.*^11^ investigated the oxidative desulfurization using titanium-modified SBA-16, showing that it could achieve 90% of sulfur removal from a 0.2 wt% DBT solution at 60 °C in less than 1 h by using H_2_O_2_. Qiu *et al.*^12^ studied the oxidative desulfurization performance of molybdenum supported on modified medicinal stone (Mo/MMS). The removal rate of DBT reached 98.1% at 103 °C through oxidation treatment. Zhao *et al.*^13^ reported the performance of extractive desulfurization using *N*,*N*-dimethylacetamide (DMAC), *N*,*N*-dimethylformamide (DMF) and tetramethylenesulfone (TMS) mixed solvent. The extraction of DBT reached 99.1% at optimal conditions. However, the oxidants and solvents used in ODS and EDS are expensive, and exhibit some potential safety hazards. Therefore, developing new methods for removing the sulfur in fuels is a persistent challenge faced by researchers.

Adsorptive desulfurization (ADS) is considered as one of the most promising methods to obtain ultra-low sulfur fuels.^14,15^ This process has several advantages over conventional HDS processes. ADS can be carried out at atmospheric pressure and room temperature, without consumption of hydrogen.^3,16–18^ Various high performance materials have been reported as adsorbents for adsorptive desulfurization of fuels. The most commonly-used adsorbents include activated carbons,^19–21^ zeolites,^22–24^ ionic liquids,^25–27^ metal oxides^28–30^ and other mesoporous materials.^31^ Bazyari *et al.*^32^ investigated the TiO_2_–SiO_2_ nanocomposite catalyst-adsorbents. This catalyst with 50 wt% TiO_2_ (TS-50) exhibited the highest ODS activity, achieving more than 98% sulfur removal for less than 10 ppm sulfur in model fuel (2875 ppm DBT in isooctane) in 20 min. In addition, TiO_2_–SiO_2_ complex oxides have considerably high specific surface areas and surface acidities, which can significantly improve the desulfurization performance of ADS. Xu *et al.*^3^ examined the adsorptive desulfurization performance of NiO–CeO_2_/Al_2_O_3_–SiO_2_ adsorbents and demonstrated that it could approach a sulfur adsorption capacity of 3.22 mg g^−1^ at breakthrough points of 50 ppm in the Jet-A fuel at ambient conditions. Miao *et al.*^33^ studied selective adsorption of thiophenic compounds from fuel over TiO_2_/SiO_2_ under UV-irradiation and found that a high sulfur adsorption capacity of 5.12 mg g^−1^ was obtained at a low sulfur concentration of 15 ppm. The involved ADS mechanisms were elucidated as π-complexation, S–M chemisorption, S–H interactions, H-bonding interactions and van der Waals interactions.^34–37^

It was also pointed out that specific surface area and surface acidity in the adsorption process could influence the overall adsorption performance of different types of adsorbents. According to Lewis acid–base theory, most thiophene-based sulfur compounds in commercial fuels appear to be Lewis bases, which are easily adsorbed by Lewis acidic sites.^38^ In this study, the desulfurization adsorbent was designed based on Tanabe's hypothesis^39^ (see ESI† Section S1). TiO_2_–SiO_2_ binary oxides were used as adsorbents and the effect of TiO_2_/SiO_2_ mass ratios on the performance was tested systematically. The unique surface effects of TiO_2_ ensured good low-temperature desulfurization activity, but its thermal stability and mechanical stability were poor. It has been reported that the thermal stability and the crystalline stability of TiO_2_ could be remarkably enhanced by SiO_2_ modification. Moreover, the surface acidity and inter-atomic interaction of TiO_2_–SiO_2_ complex oxides has a direct relationship with the mass ratio of TiO_2_/SiO_2_. The amount of Lewis acid would increase when TiO_2_ was modified by SiO_2_, and the Bronsted acid center was produced at the same time, which could promote the desulfurization performance. Compared with hydrodesulfurization, TiO_2_–SiO_2_ complex oxides-based adsorbents can achieve deep desulphurization at room temperature and atmospheric pressure without consuming hydrogen. Compared with oxidative desulfurization and extraction desulfurization, the adsorption desulfurization process can operate without oxidants, thus reducing the cost and improving the stability. Furthermore, the TiO_2_–SiO_2_ adsorbent can be easily regenerated.

## 2. Experimental section

### 2.1. Materials preparation

Tetraethylorthosilicate (TEOS, ≥99%), tetraethyltitanate (TBT, 95%), ethanol (EtOH, ≥99%), nitric acid (HNO_3_, 69 wt%), benzothiophene (BT, ≥99%), dibenzothiophene (DBT, ≥98%), and isooctane (≥99%) were all obtained from Merck. All chemicals were used without further purification.

#### 2.1.1. Model fuel

Quantitative amounts of BT, BT&DBT, and DBT were dissolved in isooctane solvent, such that the sulfur contents of the model fuel samples were 500 ppm, 500 ppm (250 ppm BT + 250 ppm DBT) and 550 ppm, respectively.

Mesoporous TiO_2_–SiO_2_ complex oxides were prepared by a sol–gel method, which is an effective way to synthesize homogeneous metal oxide materials. The preparation process was as follows: an appropriate amount of ethanol was first added to a 250 mL beaker; then, appropriate amounts of TBT and TEOS were introduced, followed by stirring at room temperature for 5 min (liquid A). A mixture of ethanol : H_2_O : HNO_3_ with a molar ratio of 10 : 4 : 1 in a second beaker was stirred for 5 min (liquid B). Liquid B was then added dropwise to liquid A and stirred at 30 °C for half an hour. The reactor was then gradually heated to 80 °C. The mixed solution was further stirred for several hours until a wet sol was obtained and the temperature was maintained for another two hours. The prepared wet sol was dried at 100 °C in a blast oven, followed by calcination at 500 °C, 550 °C, 600 °C, and 650 °C for 3 h in a muffle furnace before further use. The adsorbent TiO_2_–SiO_2_ complex oxides were pure TiO_2_, Ti–Si-20 (20 wt% SiO_2_), Ti–Si-40 (40 wt% SiO_2_), Ti–Si-60 (60 wt% SiO_2_), Ti–Si-80 (80 wt% SiO_2_) and pure SiO_2_.

### 2.2. Materials characterization

Scanning electron microscopy (SEM) was conducted on a JEOL JMS-5900 apparatus to observe the microstructure of the sample. A 15 kV acceleration voltage was used to determine the morphology and particle size of the sample. The phase structures of the samples were obtained by an X-ray diffractometer (Smartlab TM 3Kw, Rigaku, Japan) using Cu Kα radiation. The 2*θ* scans covered the range 10–80° and the accelerating voltage and applying current were 40 kV and 40 mA, respectively. Pore size distribution was calculated by the Barrett–Joyner–Halenda (BJH) method from the adsorption branch of the N_2_ physisorption isotherms. All of the samples were degassed at 350 °C under vacuum for 3 h prior to the adsorption experiments. The surface functional groups of the adsorbents were measured by Fourier transform infrared spectra (FT-IR) of NH_3_ adsorption. The surface acidity of the adsorbents were evaluated by temperature programmed desorption (TPD) of ammonia using CHEMBET-3000 (Quantachrome).

### 2.3. Desulfurization activity testing

All experiments were performed under atmospheric pressure and room temperature using static saturation tests, in which 5 g of model fuel (DBT) was mixed with 0.2 g of adsorbent and ultrasonically treated for 10 min (to improve mass transfer efficiency). The residual sulfur concentration of the model fuel was analyzed every 20 min for a period of 3 h to determine the equilibrium adsorption capacity. The saturated sulfur capacity was also measured. In this test, 0.3 g of adsorbent was mixed with 10 g of model fuel in a glass spawn bottle. The mixture was first ultrasonically treated for 10 min and then kept static for 12 h. The saturated sulfur capacity was calculated based on the sulfur concentrations before and after adsorption. The total sulfur concentration was measured by a KHWLS-200 total sulfur analyzer with a working range of 0.2–10 000 ppm and an uncertainty of less than 5% of the measured value. Each sample was analyzed to calculate the corresponding desulfurization efficiency and saturated sulfur capacity according to eqn (1) and (2):1
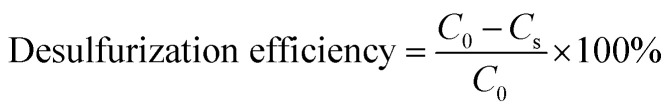
2
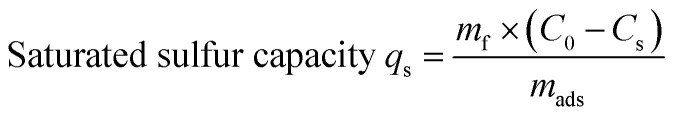
where *C*_0_ is the initial sulfur concentration (ppm), *C*_s_ is the residual sulfur concentration (ppm), *m*_f_ (gram) is the mass of fuel sample, and *m*_ads_ (gram) is the mass of the adsorbent.

**Fig. 1:**
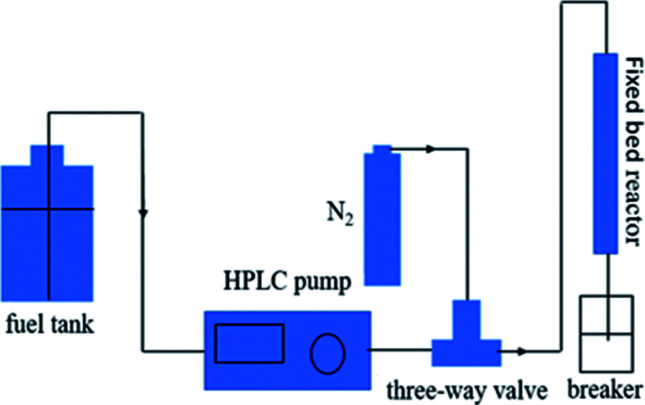
Schematic of the fixed-bed sulfur adsorption system.

Dynamic breakthrough experiments were carried out using a self-designed fixed-bed test. For each test run, 2 g of pre-weighted adsorbent was put in a stainless tube containing model fuel with a measured initial sulfur concentration of 550 ppm at room temperature and atmospheric pressure. To improve the quality of results, the amount of adsorbent was increased by tenfold compared to the static saturation tests. The fuel flowed vertically upward at a constant flow rate of 0.1 mL min^−1^. The corresponding liquid hourly space velocity (LHSV) was 4 h^−1^. The schematic of the fixed-bed sulfur adsorption system is shown in Fig. 1. The desulfurization performances of the adsorbents were characterized by measuring the residual sulfur concentration in the fuel. The breakthrough curves were obtained by plotting the instantaneous sulfur concentration and the initial sulfur concentration by the mass of adsorbent used in this study. The breakthrough sulfur capacity is defined according to (eqn (3)):3
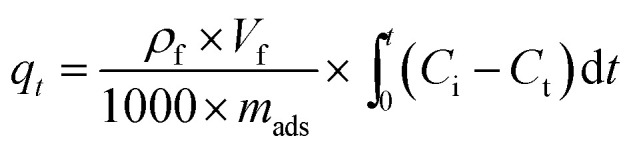
where *C*_0_ is the initial sulfur concentration (ppm), *C*_t_ is the instantaneous sulfur concentration (ppm), *ρ*_f_ (g mL^−1^) is the density of fuel, *V*_f_ (mL min^−1^) is the fuel flow rate, and *m*_ads_ (gram) is the mass of the adsorbent, which includes the adsorbents and support material.

As the adsorption reaction proceeds, the adsorbents turned gradually yellow and became saturated as the content of adsorbed thiophene increased. Spent adsorbents can be regenerated by heating at high temperature. The detailed steps are as follows: the spent adsorbent was washed with ethanol and ultrasonically treated for 5 min; then, it was dried at 100 °C for 30 min and reactivated by high temperature calcination at 550 °C for 2 h. The desulfurization performance of the regenerated adsorbent was measured in a new reaction.

## 3. Results and discussion

### 3.1. Effect of titania/silica mass ratio on the ADS performance

**Fig. 2:**
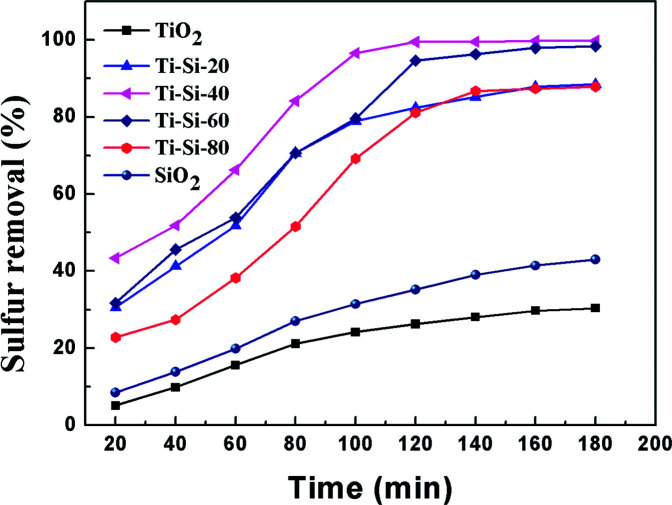
Adsorption efficiency of adsorbents with different titania and silica mass ratios over time in static saturation tests (5 g of model fuel (550 ppm DBT in isooctane), 0.2 g of adsorbents).

The results of the adsorbents with different titania and silica mass ratios in static saturation tests are shown in Fig. 2. Each of these adsorbents was measured for ADS of the model fuel at room temperature for 3 h. It can be observed that the desulfurization performance of the titania–silica complex oxides was significantly better than that of pure titania or silica. When the silica mass fraction is 40 wt%, the desulfurization efficiency of the Ti–Si-40 reaches 99%. During the initial two hours of the reaction, the desulfurization rates increased in the following order: pure titania < pure silica < Ti–Si-20 < Ti–Si-80 < Ti–Si-60 < Ti–Si-40. This suggested that the TiO_2_–SiO_2_ complex oxides were the key components for adsorptive desulfurization. In order to certify that the TiO_2_–SiO_2_ adsorbent was not a simple physical mixture, titania and silica powder adsorbent was prepared by extrusion. The desulfurization performance of the extruded powder was measured and the results are shown in Section S2.†

**Fig. 3:**
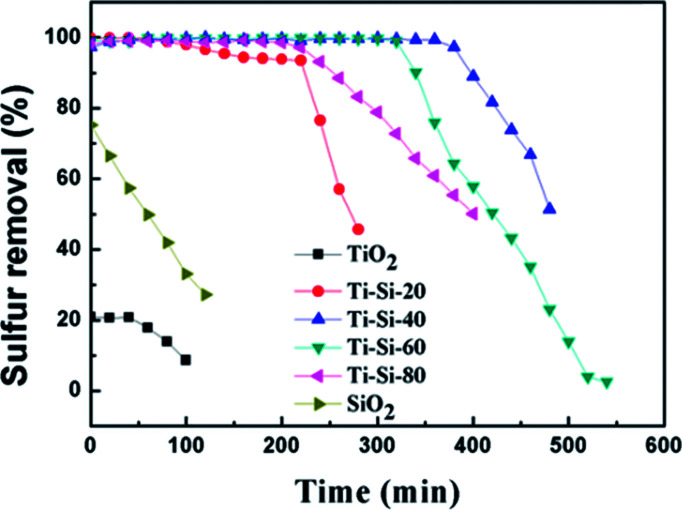
Adsorption efficiency of adsorbents with different titania and silica mass ratios over time in fixed-bed tests (model fuel (550 ppm DBT in isooctane), 2 g of adsorbents and LHSV = 4 h^−1^).

Fig. 3 shows the breakthrough curve of the TiO_2_–SiO_2_ complex oxides for DBT at a LHSV of 4 h^−1^. For the Ti–Si-40 adsorbent, the highest breakthrough capacity calculated for DBT was 7.1 mg g^−1^ and the life time was 380 min. In static saturation tests, the saturated sulfur capacity in the case of DBT could reach 13.7 mg g^−1^, which was over 51.5% in comparison with the breakthrough sulfur capacity. All of the TiO_2_–SiO_2_ adsorbents were more efficient and have higher sulfur removal rates than silica or titania for the ADS reaction. It was found that the mesoporous TiO_2_–SiO_2_ adsorbent had a significantly improved sulfur capacity compared with traditional adsorbents.^3,29^ In addition, compared with ODS and EDS, TiO_2_–SiO_2_ adsorbents can maintain high desulfurization performance at ambient conditions without oxidative treatment.^8–13^ Therefore, this adsorbent is a potential industrial desulfurization material.

**Fig. 4:**
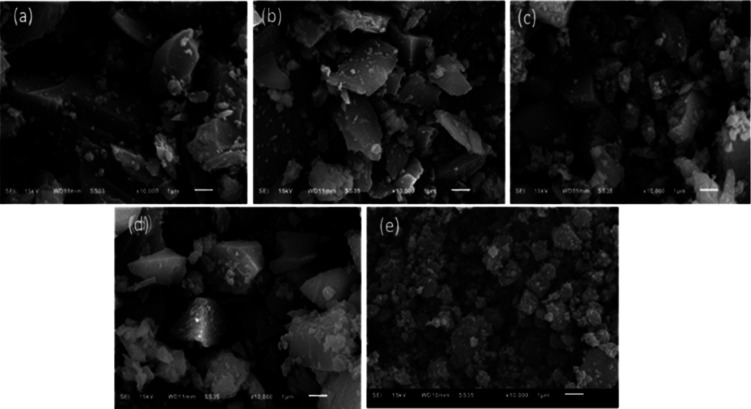
SEM photographs of Ti–Si-20 (a), Ti–Si-40 (b), Ti–Si-60 (c), Ti–Si-80 (d) prepared by a sol–gel method and TiO_2_–SiO_2_ powders (e) prepared by extrusion.

In order to further study the surface morphology of the TiO_2_–SiO_2_ adsorbents, SEM technique was used. As shown in Fig. 4, all of these different TiO_2_–SiO_2_ adsorbents have irregular blocky structures that are quite similar. They are dramatically different from the surface morphologies of TiO_2_–SiO_2_ powder adsorbents prepared by extrusion, which show a porous coral-like structure.

**Fig. 5:**
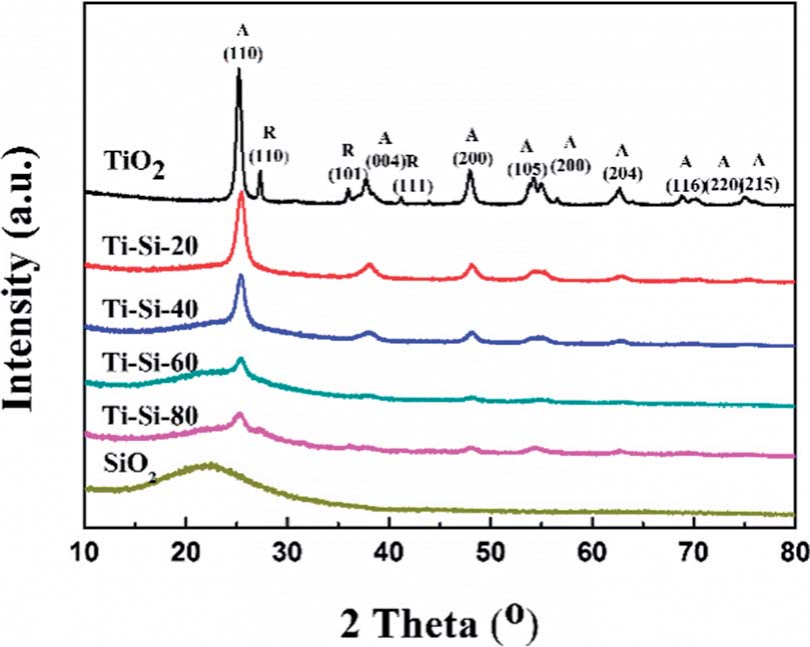
XRD characterization of adsorbents with different silica content.

Fig. 5 shows the X-ray diffraction patterns of the TiO_2_–SiO_2_ complex oxides. There are no diffraction peaks observed for the crystalline silica phase, indicating that pure SiO_2_ is amorphous. Moreover, pure titania showed typical diffraction peaks at around 25.29°, 27.65°, 38.42°, and 48.91°, which are indexed to (101), (110), (004) and (200) planes, respectively. Anatase and rutile crystalline phases also coexist simultaneously. For the TiO_2_–SiO_2_ complex oxides, only the anatase crystalline phase was observed. The rutile crystalline phase disappeared with the increase in silica content. Features typical of amorphous structures were found for TS-80 when the silica content reached 80 wt%. This confirmed that highly dispersed crystalline phases of titania on silica could be found in the TiO_2_–SiO_2_ complex oxides. In addition, the difference in intensities of peaks for various TiO_2_–SiO_2_ adsorbents indicates a variation in the corresponding amounts of each adsorbent. It can be verified that silica can largely improve the thermal stability of titania. A previous report has shown that anatase is better than rutile for sulfur adsorption from liquid fuels.^40^

It is generally known that adsorption–desulfurization performance is heavily dependent on the specific surface areas of the adsorbents. In order to investigate the microscopic surface texture, N_2_ adsorption–desorption isotherms were measured and pore size distribution for TiO_2_–SiO_2_ complex oxides was calculated (see Table 1). All of the samples were calcined at 600 °C. As indicated by the data in Table 1, the surface areas were strongly dependent on the silica content and increased from 66 m^2^ g^−1^ (pure titania) to 490 m^2^ g^−1^ (pure silica). Considering the raw material composition, TiO_2_–SiO_2_ adsorbents could achieve and maintain large surface areas on addition of silica component. In addition, it has been confirmed that direct sulfur-adsorbent interaction plays an important role in the adsorptive desulfurization process.^41^ Ti–Si-40 exhibited both the highest desulfurization rate and capacity, according to previous test results. The specific surface area and pore size of the Ti–Si-40 was 315 m^2^ g^−1^ and 4.8 nm, respectively. Despite having the largest specific surface area of 490 m^2^ g^−1^, the desulfurization efficiency of pure silica only reached 42%. This indicates that TiO_2_–SiO_2_ complex oxides were the main active component and not the pure silica. Both the Ti–Si-60 and Ti–Si-80 adsorbents had larger specific surface areas than did the Ti–Si-40 adsorbent. However, their desulfurization efficiencies were still less than that of the Ti–Si-40 complex oxide. These results indicated that specific surface area was not the only factor influencing the desulfurization performance and that it is dominated by other factors.

**Table 1:** Physical properties of titania–silica adsorbents

Adsorbent	SiO_2_ (wt%)	Surface area (m^2^ g^−1^)	Average pore size (nm)	Total pore volume (cm^3^ g^−1^)
TiO_2_	0	66.3	7.9	0.13
Ti–Si-20	20	157.1	5.5	0.22
Ti–Si-40	40	315.4	4.8	0.28
Ti–Si-60	60	337.7	2.6	0.22
Ti–Si-80	80	461	2.2	0.27
SiO_2_	100	489.7	2.0	0.24

**Fig. 6:**
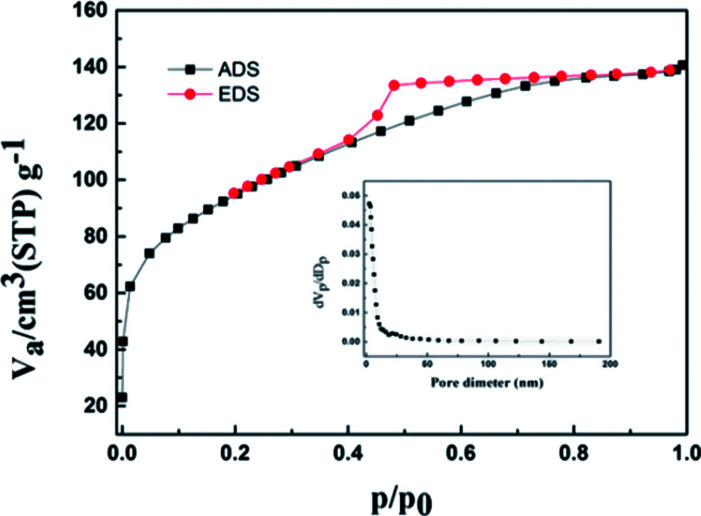
N_2_ adsorption–desorption isotherms and corresponding pore size analysis of Ti–Si-40.

Fig. 6 illustrates the N_2_ adsorption–desorption isotherms for the Ti–Si-40 complex oxide, which are in accordance with isotherm I. This indicated that the microstructure of Ti–Si-40 is large enough to accommodate DBT molecules and other bulky sulfur compounds. The critical diameters of the DBT molecules were less than 1 nm, which are smaller than the pores size of the Ti–Si-40 adsorbent. Thiophene and its derivatives could easily diffuse into the pores, where most of the active sites for ADS adsorption were located.

**Fig. 7:**
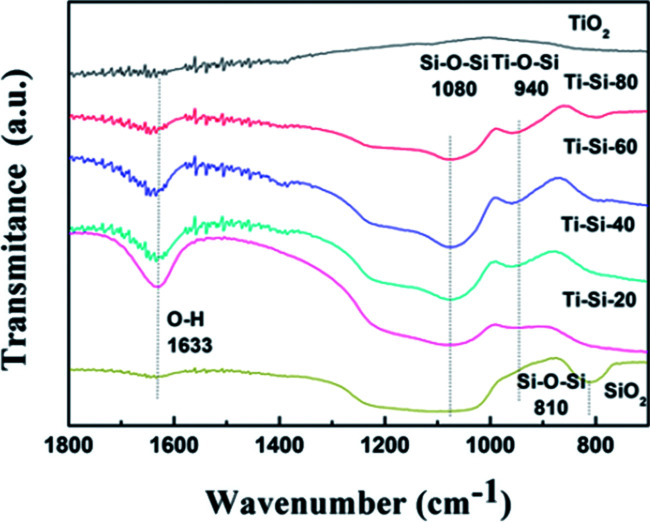
FTIR spectrum of the silica, titania and TiO_2_–SiO_2_ adsorbents prepared in this study.

Fig. 7 presents the FT-IR spectra of pure titania, TiO_2_–SiO_2_ complex oxides, and pure silica. According to the related literature analysis,^42^ the peaks appearing at 810–800 cm^−1^, 1105–1080 cm^−1^, 960–910 cm^−1^ and 1650–1620 cm^−1^ correspond to the symmetric stretching of Si–O–Si, asymmetric Si–O–Si vibration, stretching vibration of Ti–O–Si and bending vibration of OH groups, respectively. The band appearing at 940 cm^−1^ demonstrates the successful incorporation of titanium into the silica framework for the TiO_2_–SiO_2_ complex oxides. The peak becomes more intense with the increase in Si content, clearly indicating that changing the silica content can influence the atomic scale structure of the TiO_2_–SiO_2_ adsorbents. It can be speculated that the formation of Ti–O–Si linkages are crucial to the desulfurization performance of different TiO_2_–SiO_2_ binary oxides.

**Fig. 8:**
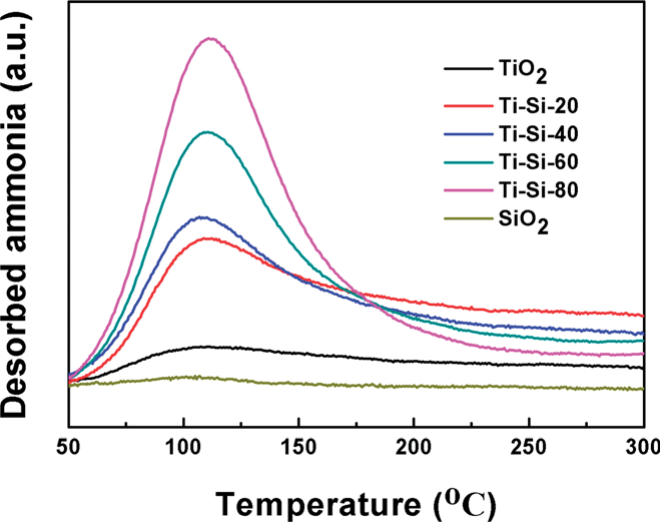
NH_3_-TPD profiles of silica, titania and TiO_2_–SiO_2_ adsorbents.

It has been reported that the surface acidity of adsorbents can play an important role in the adsorption capacity of thiophene and its derivatives.^16^ NH_3_-TPD is a common method for analyzing surface acidity. Fig. 8 illustrates the NH_3_-TPD profiles of pure silica, pure titania and TiO_2_–SiO_2_ complex oxides. The broad desorption peaks spanning 100–150 °C are attributed to the weak acid sites. Pure silica and titania have slight acidity, which are easily desorbed, resulting in their poor desulfurization performances. On the contrary, the acidic properties of TiO_2_–SiO_2_ complex oxides were quite different from those of titania and silica. With the increase in TiO_2_ content, the number of acidic sites increased monotonously, which can be ascribed to an increase in exposed Ti species. According to Tanabe's hypothesis, a binary oxide with TiO_2_ as the major oxide component exhibits Lewis acidity. Therefore, the large specific surface area and the Lewis acidity of mesoporous TiO_2_–SiO_2_ binary oxides will remarkably promote adsorption capacity of thiophene sulfurs and its derivatives at low temperatures. However, the complex oxide exhibited the Brønsted acidity or Lewis base when SiO_2_ was the main component oxide, which repulses the thiophene sulfur.^43^ These results are in accordance with the results obtained in the previous experiment.

### 3.2. Effect of calcination temperature

**Fig. 9:**
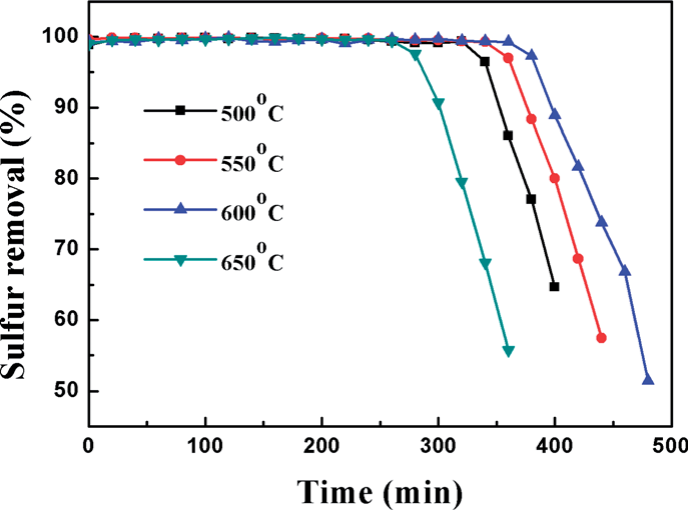
Dependence of DBT removal *vs.* reaction time on the calcination temperature of Ti–Si-40.

In order to study the effect of calcination temperature of the titania–silica on the desulfurization performance, the Ti–Si-40 adsorbent was calcined at 500 °C, 550 °C, 600 °C and 650 °C. The relationship between the desulfurization results and the reaction time is presented in Fig. 9 and Table 2. It can be inferred that the calcination temperature significantly affects the performance of TiO_2_–SiO_2_ complex oxides as the desulfurization performance increased with temperature at first and then decreased after the calcination temperature was higher than 600 °C. High-temperature processing could remove water and improve the adsorbent surface area, which is supposed to greatly promote the adsorption reaction.^44^ In addition, it can be concluded that high-temperature process can lead to the generation of adsorption centres on the TiO_2_–SiO_2_ complex oxides. However, further increasing the calcination temperature would have a negative impact on the desulfurization performance because excessive calcination temperature can decrease the surface area and reduce surface adsorption site density. On the contrary, lower calcination temperatures are not adequate to completely decompose the organic residues in Ti–Si-40, which could block surface adsorption sites. According to previous research, thiophene and its derivatives are Lewis bases. Therefore, they can be adsorbed on the adsorption sites of TiO_2_–SiO_2_*via* a Lewis acid–base interaction.^45–47^ This acid–base interaction can facilitate the adsorption of DBT on the surface of adsorbents, which improves the ADS efficiency.

**Table 2:** Effect of calcination temperature on sulfur removal performance

Parameters	Total S contents (550 ppm)			
Calcination temperature (°C)	*V*_f_ (mL min^−1^)	*m*_ads_ (g)	Capacity (mg g^−1^)	Life time (min)
500	0.1	2	6.3	340
550	0.1	2	6.9	370
600	0.1	2	7.8	380
650	0.1	2	5.4	290

### 3.3. ADS selectivity of different sulfur compounds

**Fig. 10:**
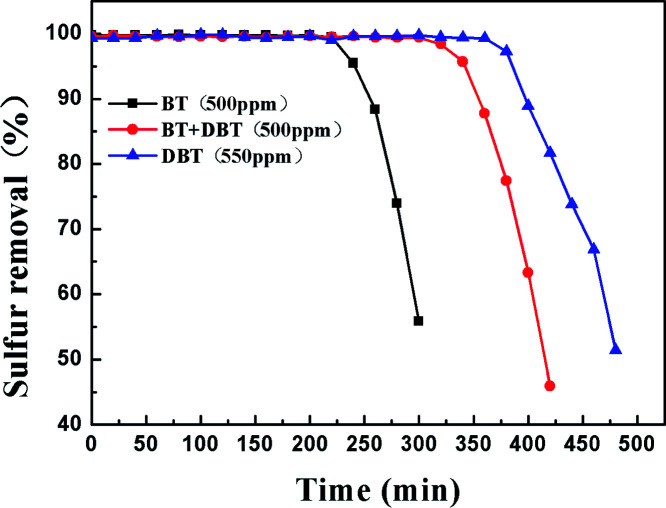
Reaction times for the removal of different sulfur compounds over Ti–Si-40 (model fuel, 2 g adsorbents and LHSV = 4 h^−1^).

In order to investigate the adsorption selectivity of different refractory sulfur compounds, model fuels composed of 500 ppm BT, 500 ppm BT&DBT (250 ppm BT + 250 ppm DBT) and 550 ppm DBT were prepared using isooctane. The test results are shown in Fig. 10. It can be observed that both DBT and BT were completely removed at room temperature and atmospheric pressure. BT exhibited the lowest breakthrough sulfur capacity and its lifetime was only 200 min. The adsorption selectivity for the different sulfur compounds increased in the order of BT < BT&DBT < DBT. It has been reported that electron density on sulfur atoms and steric hindrance may play important roles in adsorption desulfurization.^48^ The electron density of DBT and BT are 5.758 and 5.696,^49^ respectively. According to their electron density, DBT is much easier to adsorb on the surface of the adsorbent. It can be inferred that adsorption selectivity is sensitive to the electron density on sulfur atoms.

### 3.4. Kinetics of the ADS

In order to investigate the adsorption process, classic kinetic models were examined to describe the sulfur adsorption kinetics of the TiO_2_–SiO_2_ complex oxide adsorbents. All kinetic models were assumed to predict the reaction rate in the absence of mass transfer limitations.

An empirical kinetic model^32^ was expressed as follows:4
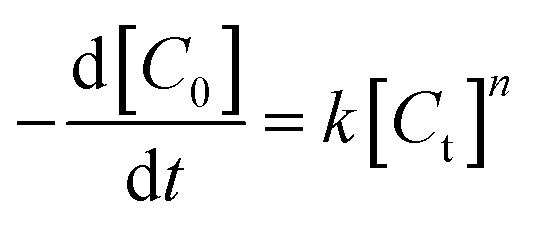
where *k* is the apparent rate constant and *C*_0_ and *C*_t_ are the initial and instantaneous concentrations of DBT respectively. Eqn (4) has been integrated for *n* = 1; the integrated form of eqn (4) is given as:5
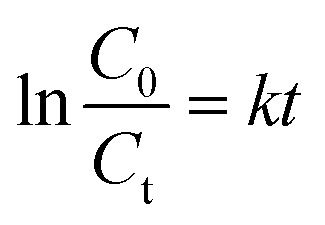


**Fig. 11:**
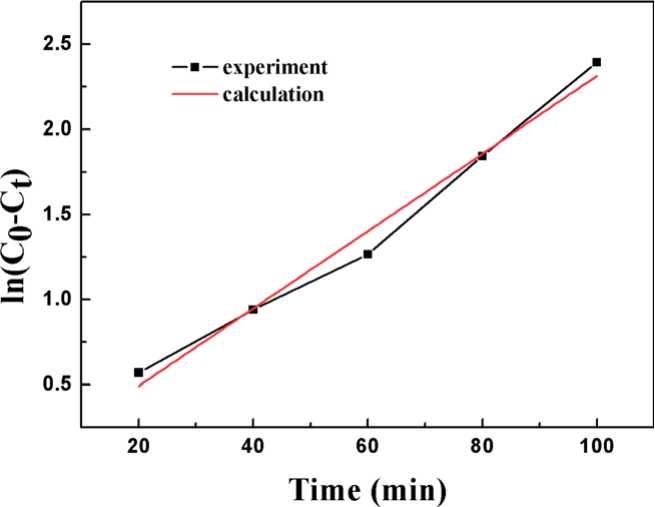
Kinetic model analysis: an empirical kinetic model.

Fig. 11 shows the empirical ADS kinetics of DBT over the Ti–Si-40 adsorbent. It can be noticed that a good linear relationship is obtained between ln(*C*_0_ − *C*_t_) and reaction time. The correlation coefficient was over 0.98, indicating that the adsorption process can be well-expressed by this empirical kinetics model. In other words, the adsorption desulfurization reaction follows pseudo-first order kinetics.

The pseudo-second order model^50,51^ is presented as follows:

6 *r*_q_ = *k*_s_(*q*_e_ − *q*_*t*_)^2^ = d*q*_*t*_/d*t*

where *q*_*t*_ indicates the amount of sulfur adsorbed per unit mass of adsorbent in a certain period of time, *r*_q_ indicates the sulfur adsorption rate, *k*_s_ indicates the rate constant and *q*_e_ indicates the amount of sulfur adsorbed per unit mass of adsorbent at equilibrium state, which is around 13.7 mg g^−1^ according to the current experimental results. The integrated form of eqn (7) yields:7
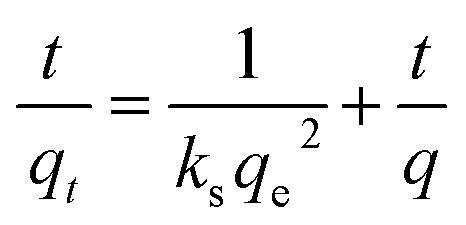


The intra-particle diffusion (IPD) model proposed^46^ is expressed in the form of:

8 *q*_*t*_ = *k*_i_*t*^1/2^ + *C*

where *k*_i_ indicates the intra-particle diffusion model rate constant and *C* indicates a constant.

**Fig. 12:**
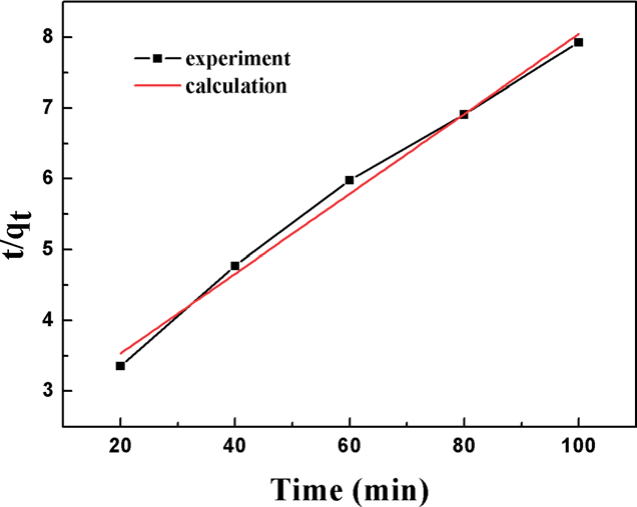
Kinetic model analysis: pseudo-second order model.

**Fig. 13:**
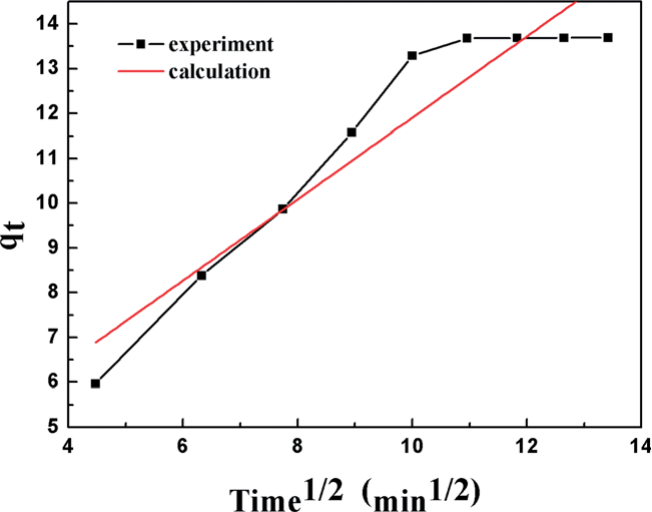
Kinetic model analysis: intra-particle diffusion model.

The results of the pseudo-second order kinetic model are shown in Fig. 12 and 13. It can be observed that this model perfectly fits the experimental data. There is a linear relationship between *t*/*q*_*t*_ and reaction time for adsorption desulfurization as shown in Fig. 12. The correlation coefficient and other parameters of the three kinetic models are listed in Table 3. It can be observed that the pseudo-second order model has a high correlation coefficient (0.99), which illustrates that the TiO_2_–SiO_2_ adsorbent could be well described by this model. The intra-particle diffusion model was applied to further measure the adsorption of DBT. As shown in Fig. 13, the plot of *q*_*t*_*versus t*^1/2^ is non-linear. This indicates that two or more steps occur in the adsorption process. According to our knowledge of adsorption, the following hypothesis is proposed: in the first stage, DBT adsorbs on the adsorbent *via* physical absorption and S–H interaction. In second stage, intra-particle diffusion controls the overall adsorption rate.

**Table 3:** Kinetic analysis results of two different models

		Correlation coefficient (*R*^2^)
**Pseudo-second order model**		
*q*_e,cal_ = 13.6 (mg g^−1^)	*k*_f_ = 0.056 (min^−1^)	0.99

**Intra-particle diffusion model**		
*C* = 8.19 (mg g^−1^)	*k*_i_ = 0.909 (mg g^−1^ min^−1/2^)	0.8926

### 3.5. Regeneration of adsorbent

**Fig. 14:**
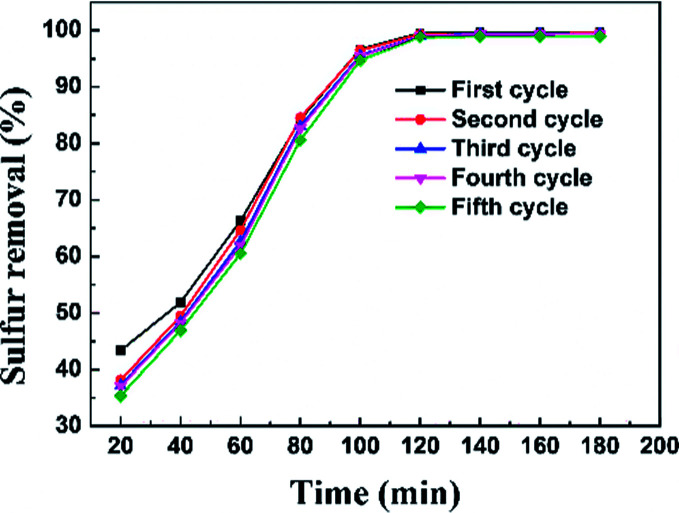
Sulfur removal efficiency of Ti–Si-40 adsorbent over five regeneration cycles.

**Fig. 15:**
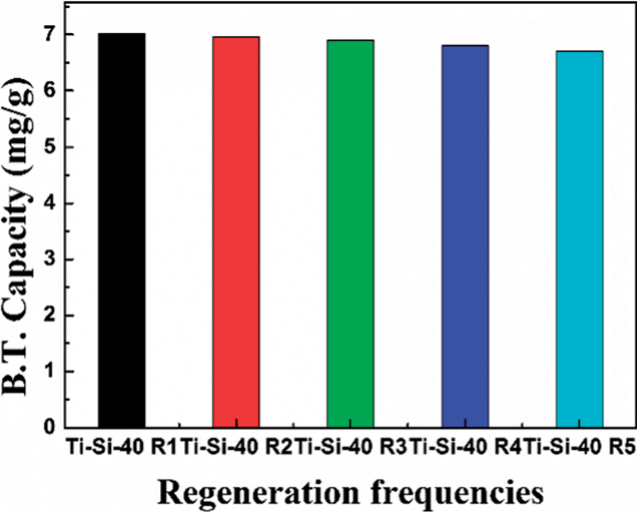
Breakthrough capacity of Ti–Si-40 adsorbent over five regeneration cycles.

The regeneration of an adsorbent is one of the most important practical issues for industrial application. A series of regeneration tests were conducted and the results are shown in the Fig. 14 and 15. The regenerative process of the spent Ti–Si-40 adsorbents is discussed above (see in desulfurization experiments). The preliminary tests of adsorbent regeneration showed that the first-time regenerated adsorbent could recover 99% of the breakthrough capacity as compared to a fresh adsorbent and after the fifth regeneration, it could recover 94.5%. This result confirms that the majority of adsorption desulfurization capacity can be recovered after quintic regeneration.

## 4. Conclusions

Mesoporous titania–silica adsorbents were synthesized for ultra-deep adsorption desulfurization (ADS) of model fuel in static adsorption experiments using a fixed-bed reactor at room temperature and ambient pressure. Increasing the silica content improved the thermal stability of titania and increased the specific surface area of titania–silica complex oxides. Moreover, titania–silica binary oxides exhibited a stronger surface acidity than pure titania and pure silica, thus significantly improving the desulfurization performance. In the fixed-bed tests, the highest measured sulfur adsorption capacity was 7.1 mg g^−1^ for the Ti–Si-40 adsorbent. In the static adsorption experiments, the saturated sulfur capacity reaches 13.7 mg g^−1^.

The lager specific surface area and the stronger Lewis acidity of the mesoporous TiO_2_–SiO_2_ binary oxide remarkably enhanced adsorption capacity of thiophene sulfurs and its derivatives at low temperatures. Kinetic studies showed that the pseudo-second order model could be used to describe the adsorption kinetics satisfactorily. The adsorbent could be regenerated by high temperature treatment with negligible loss in activity, indicating that the mesoporous titania–silica adsorbents are highly stable. The desulfurization experiment for crude oil will be carried out in the future.

## Conflicts of interest

There are no conflicts to declare.

## Supplementary Material

RA-008-C8RA00112J-s001
