# Supplementary material for: Mesoporous TiO2–SiO2 adsorbent for ultra-deep desulfurization of organic-S at room temperature and atmospheric pressure
Source: RSC Adv. 2018 Feb 16;8(14):7579–87. doi: 10.1039/c8ra00112j (PMC9078429; doi:10.1039/c8ra00112j)
Supplement: RA-008-C8RA00112J-s001 [file RA-008-C8RA00112J-s001.pdf]

Electronic Supplementary Material (ESI) for RSC Advances.
This journal is © The Royal Society of Chemistry 2018

Supplementary Material

Mesoporous TiO_2-SiO_2 adsorbent: ultra-deep desulfurization of organic-S at room temperature and atmospheric pressure

Bin Qin [a, b, c], Yuesong Shen [a, b, c,*], Boyang Xu [a, b, c], Shemin Zhu [a, b, c], Peiwen Li [d] *and Youlin liu* [a, b, c]

[a] *College of Materials Science and Engineering,* [b] *Jiangsu Collaborative Innovation Center for Advanced Inorganic Function Composites,* [c] *Jiangsu National Synergetic Innovation Center for Advanced Materials, Nanjing Tech University, No.5 Xinmofan road, Nanjing 210009, China*

[d] *Department of Aerospace and Mechanical Engineering, University of Arizona, Tucson, AZ, 85721-0119, USA*

***Corresponding authors:** shenyuesong@njtech.edu.cn (Yuesong Shen)

Section S1: Tanabe's hypothesis

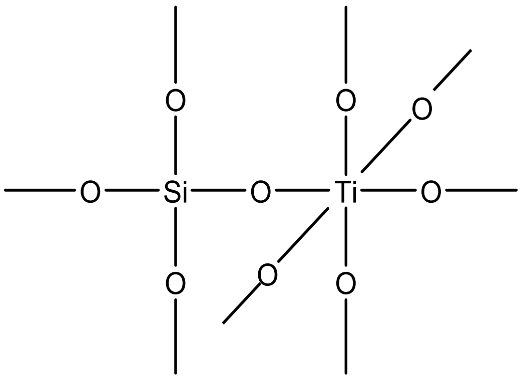

(a) charge difference= （+4/6-2/2） *6=-2

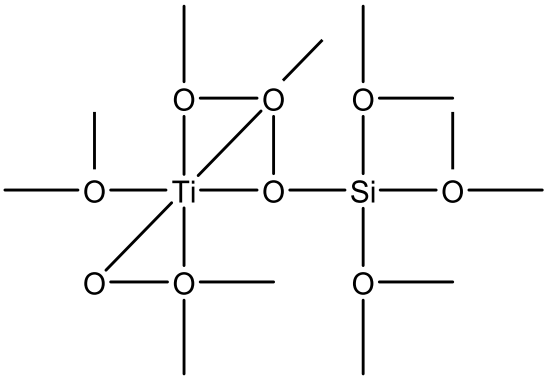

(b) charge difference = （+4/4-2/3） *4= +4/3

Fig. S1. Model structures of TiO_2–SiO_2 and SiO_2–TiO_2 pictured according to Tanabe's hypothesis. (a) with SiO_2 as the major oxide; (b) with TiO_2 as the major oxide.

The SiO_2 is major component oxide in the structure of TiO_2-SiO_2, which is shown in Figure.S1 (a). According to Tanabe's hypothesis the charge difference for each bond is (+4/6-2/2) = -1/3, and for all the bonds the valence unit is (+4/6-2/2) * 6 = -2. In this case, the Brønsted acidity is assumed to appear in the presence of an excess of the negative charge, because three protons are considered to be associated with six oxygens to keep the electric neutrality. Although the TiO_2-SiO_2 adsorbent exhibits big specific surface area and showed good performance, the TiO_2-SiO_2 binary oxide itself exhibits the Brønsted acidity or Lewis base which is not benifical for adsorbing sulfur at low temperatures. However, if the binary oxide mode was changed into TiO_2-SiO_2 with TiO_2 being the major component, the result will be contrary for

effect. The structure of TiO_2-SiO_2 with TiO_2 being the major component oxide is shown in Figure.S1 (b). According to Tanabe's hypothesis, the charge difference for each bond is (+4/4-2/3)=+1/3, and for all the bonds the valence unit of(+4/4-2/3)*4=+4/3 is excess. In this case, the Lewis acidity is assumed to appear in the presence of an excess of the positive charge. Thereby the adsorption capacity of thiophene sulfurs and their derivatives would be significantly enhanced at low temperatures owing to high specific surface and rich Lewis acidity of the mesoporous TiO_2-SiO_2 binary oxide.

Section S2: The effect of preparation method on desulfurization performance

Figure.S2 give a clear comparison of the desulfurization efficiencies of Ti-Si-40 adsorbent by different preparation methods in the same conditions. As is can be seen, the titania and silica powder adsorbent cannot meet the deep desulphurization requirement by simple extrusion, confirmed that TiO_2-SiO_2 complex oxides can generated adsorption sites by sol-gel.

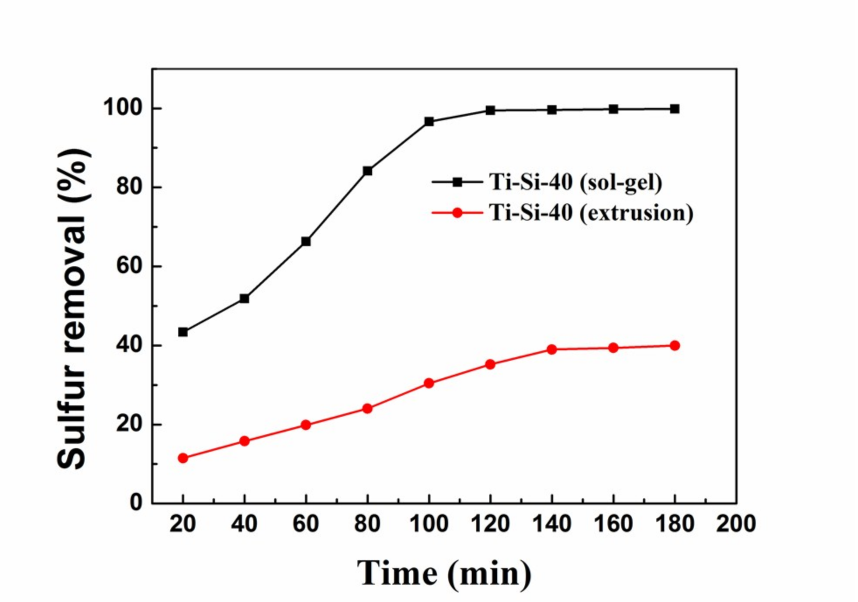

Fig. S2. Adsorption efficiency of Ti-Si-40 adsorbent by different preparation methods
